# Effects of Avelumab Maintenance on Advanced Urothelial Carcinoma: A Real‐World Multicenter Study

**DOI:** 10.1002/cam4.71241

**Published:** 2025-09-12

**Authors:** Noritaka Ishii, Yuya Sekine, Masanao Shinohara, Yohei Kawashima, Kanami Mori, Mizuki Kobayashi, Kazuyuki Numakura, Jotaro Mikami, Naoki Fujita, Teppei Okamoto, Takahiro Yoneyama, Ryuji Tabata, Satoshi Sato, Tomonori Habuchi, Chikara Ohyama, Shingo Hatakeyama

**Affiliations:** ^1^ Department of Urology Hirosaki University Graduate School of Medicine Hirosaki Japan; ^2^ Department of Urology Aomori Rosai Hospital Hachinohe Japan; ^3^ Department of Urology Akita University Graduate School of Medicine Akita Japan; ^4^ Department of Urology Ageo Central General Hospital Ageo Japan; ^5^ Department of Urology Mutsu General Hospital Mutsu Japan; ^6^ Department of Urology Tsugaru General Hospital Goshogawara Japan; ^7^ Department of Advanced Transplant and Regenerative Medicine Hirosaki University Graduate School of Medicine Hirosaki Japan

**Keywords:** avelumab, chemotherapy, immunotherapy, overall survival, urothelial carcinoma

## Abstract

**Objectives:**

Oncological outcomes in patients with urothelial carcinoma treated with avelumab maintenance therapy or conventional platinum‐based first‐line chemotherapy were compared in real‐world practice.

**Methods:**

Outcomes in patients with advanced urothelial carcinoma treated with platinum‐based first‐line chemotherapy without avelumab (chemo group, *n* = 300) or avelumab maintenance therapy (avelumab group, *n* = 85) between March 2004 and September 2024 were retrospectively evaluated. Overall survival (OS) in the chemo and avelumab groups was stratified by the number of cycles of first‐line chemotherapy. The primary outcome was OS among patients without progressive disease (non‐PD) at the cycle‐4 assessment (the standard‐switch cohort). The secondary outcome was OS among patients with non‐PD at the cycles‐2 to 3 assessment (the early‐switch cohort).

**Results:**

In the standard‐switch cohort (non‐PD at cycle 4), the chemo and avelumab groups comprised 122 and 47 patients, respectively; median OS was significantly longer with avelumab than with chemo (70 vs. 26 months; *p* = 0.015). In the early‐switch cohort (non‐PD at cycles 2–3), the chemo and avelumab groups comprised 104 and 35 patients, respectively; median OS was significantly longer with avelumab than with chemo (33 vs. 13 months; *p* = 0.002). A multivariable Cox regression analysis revealed that avelumab administration was significantly associated with a reduced risk of OS (hazard ratio, 0.37; *p* < 0.001). The retrospective design is a limitation of this study.

**Conclusion:**

Avelumab maintenance appeared to improve outcomes across cycles 2–3 and ≥ 4, though residual confounding cannot be excluded.

## Graphical abstract

From 2004–2024, 308 mUC patients in the AGEHA database were classified into early‐ (*n* = 139) and standard‐switch (*n* = 169) groups. Avelumab maintenance significantly improved OS versus continued chemotherapy in both strata (33 vs. 13 mo, *P* < 0.001; 70 vs. 26 mo, *P* = 0.015).

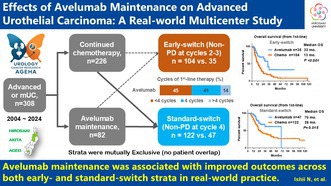

## 1 Introduction

Unresectable or metastatic urothelial carcinoma (UC) is a highly aggressive disease, typically demonstrating a median overall survival (OS) of approximately 2 years, even after treatment with platinum‐based first‐line chemotherapy followed by immune checkpoint inhibitors [1, 2, 3, 4, 5, 6, 7, 8, 9, 10, 11, 12, 13, 14, 15]. According to Level 1 evidence from the JAVELIN Bladder 100 study [16], switching to maintenance avelumab (anti‐PD‐L1 antibody) is currently the standard of care for patients without progressive disease (PD) on first‐line chemotherapy. Although multiple studies have evaluated the efficacy of avelumab, small sample sizes and short follow‐up have limited the ability to demonstrate its effectiveness in clinical practice [17, 18, 19, 20, 21, 22, 23, 24, 25, 26, 27, 28]. Furthermore, the JAVELIN Bladder 100 trial required at least 4 cycles of first‐line chemotherapy, whereas completing 4 cycles is not always feasible in clinical settings. Accordingly, the benefits of avelumab maintenance in patients who undergo an early switch (< 4 cycles) remain unclear. Moreover, a few data exist versus second‐line pembrolizumab [18, 29, 30], which was the standard of care when avelumab was approved. We therefore compared avelumab maintenance with continued platinum‐based first‐line chemotherapy in real‐world practice.

## 2 Patients and Methods

### 2.1 Study Design and Ethics Statement

This retrospective, multicenter study adhered to the tenets of the Declaration of Helsinki and was approved by the ethics committee of the Hirosaki University School of Medicine (2019‐099‐3 and 2021‐158‐2). Written consent was not obtained, but patients could choose to be excluded from the study (opt‐out approach).

### 2.2 Patient Selection and Classification

The outcomes of patients with advanced UC treated with platinum‐based first‐line chemotherapy without avelumab (chemo group, *n* = 300) or avelumab maintenance therapy (avelumab group, *n* = 85) between March 2004 and September 2024 were compared. Patients who underwent only 1 cycle of first‐line chemotherapy or patients with PD at cycle 2 were excluded. Patients were classified into four cohorts: (i) chemo, non‐PD at cycle 4 (≥ 4 cycles with disease control, no avelumab); (ii) chemo, non‐PD at cycles 2–3 (2–3 cycles with disease control, no avelumab); (iii) avelumab, non‐PD at cycle 4 (≥ 4 cycles with disease control followed by avelumab maintenance); and (iv) avelumab, non‐PD at cycles 2–3 (2–3 cycles with disease control followed by avelumab maintenance). The cycles‐2 to 3 and cycle‐4 strata were mutually exclusive (no patient overlap). For clarity, we refer to the cycle‐4 cohorts as standard‐switch and the cycles‐2 to 3 cohorts as early‐switch. Data regarding the following variables were analyzed: age, sex, Eastern Cooperative Oncology Group performance status (ECOG PS), tumor origin (urinary bladder or upper urinary tract UC), TNM stage, metastatic sites, history of local therapy (operation or radiation therapy), and cycles and types of systemic treatments. The tumor stage was stratified according to the TNM classification (8th edition). Treatment response was evaluated based on RECIST version 1.1 and classified into the following four categories: complete response, partial response, stable disease, and PD.

### 2.3 Treatment Selection

First‐line care used cisplatin‐eligible platinum regimens. Before pembrolizumab, PD was managed with second‐line chemotherapy (mainly docetaxel). After pembrolizumab approval, PD post–first‐line platinum was treated with pembrolizumab; non‐PD cases received avelumab maintenance. After enfortumab vedotin (EV) approval, PD after pembrolizumab or avelumab was typically treated with EV as third‐line therapy.

### 2.4 Early Switch Definition and Rationale

Early switch was defined as starting avelumab after 2–3 cycles of first‐line chemotherapy (before cycle 4) in the setting of non‐PD (CR/PR/SD). This decision was physician‐directed, not protocol‐driven. Although reasons were not uniformly recorded, early transition commonly reflected toxicity, performance‐status decline, comorbidities, or physician/patient preference in non‐progressive disease. For clarity, “early‐switch” and “standard‐switch” designate assessment landmarks (2–3 cycles and ≥ 4 cycles, respectively), not treatment mandates or enforced cessation at cycles 2–3.

### 2.5 Outcomes

OS was defined as the time from the initial systemic treatment to the final follow‐up or any cause of death. Disease‐free survival (DFS) was defined as the time from the initial systemic treatment to the disease progression, any cause of death, or final follow‐up.

The primary endpoint was OS among patients with non‐PD at the cycle‐4 assessment. Secondary endpoints were (i) OS among patients with non‐PD at the cycles‐2 to 3 assessment and (ii) OS comparing the avelumab and chemotherapy cohorts in the subset who subsequently received second‐line pembrolizumab. Exploratory outcomes included DFS and OS stratified by best response to first‐line chemotherapy, first‐line regimen, and number of first‐line cycles within the avelumab cohort (*n* = 85). To mitigate immortal time introduced by heterogeneity in the number/duration of first‐line cycles prior to maintenance eligibility, OS was measured from the start date of first‐line chemotherapy to death from any cause or last follow‐up.

### 2.6 Statistical Analyses

Statistical analyses were performed using BellCurve for Excel 4.07 (Social Survey Research Information Co. Ltd., Tokyo, Japan), GraphPad Prism 7.00 (GraphPad Software, San Diego, CA, USA), and R: 4.0.2 (The R Foundation, Vienna, Austria). Quantitative variables are expressed as mean ± SD or median (interquartile range [IQR]). Statistical differences were assessed using Student's *t*‐tests or the Mann–Whitney *U* tests. Categorical variables were expressed as frequencies (percentages) and compared using Fisher's exact or *χ* ^2^ tests. The OS was estimated using the Kaplan–Meier method and the log‐rank test from the initial treatment until death or final follow‐up. A multivariable Cox regression analysis was performed to assess the effects of avelumab on OS. Hazard ratios (HR) with 95% confidence intervals were calculated after controlling for potential confounders, including first‐line chemotherapy cycles < 4, ECOG PS, metastatic disease at diagnosis, treatment era before 2018, cisplatin‐based regimens, age, sex, local therapy (surgery or radiotherapy), subsequent EV therapy, and avelumab maintenance therapy. *p* values < 0.05 were considered statistically significant.

## 3 Results

### 3.1 Patient Characteristics

**FIGURE 1:**
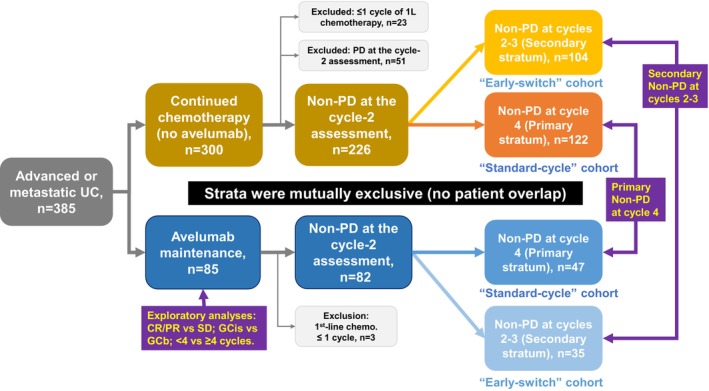
Study design and patient selection of patients. After screening 385 patients with advanced urothelial carcinoma, 26 patients with only 1 cycle of first‐line chemotherapy and 51 patients with PD at cycle 2 were excluded from the study. Patients classified as non‐PD at cycles 2–3 and those at cycle 4 constituted mutually exclusive strata with no overlap.

We identified 122 patients with non‐PD at cycle 4 in the chemo group; 104 patients with non‐PD at cycle 2–3 in the chemo group; 49 patients with non‐PD at cycle 4 in the avelumab group; and 33 patients with non‐PD at cycle 2–3 in the avelumab group (Figure 1). Those are non‐overlapping populations. The chemo and avelumab groups with non‐PD at cycle 4 included 122 and 47 patients, respectively. No significant differences in background characteristics were detected between the groups, except for the history of local therapy (39% vs. 15%, *p* = 0.003) (Table 1). The chemo and avelumab groups with non‐PD at cycle 2–3 included 104 and 35 patients, respectively. No significant differences in background characteristics were detected between the groups, except for the metastatic status (*N*+ or M1, 96% vs. 80%, *p* = 0.006) and the number of first‐line chemotherapy cycles (Table 1).

**TABLE 1:** Background of patients for primary and secondary analysis.

	Standard‐switch (non‐PD at cycle 4)		*p*	Early‐switch (non‐PD at cycles 2–3)		*p*
	Chemo	Avelumab		Chemo	Avelumab	
Number of patients	122	47		104	35	
Age, years (IQR)	72 (64, 78)	73 (67, 80)	0.086	71 (63, 77)	71 (64, 81)	0.569
Male, *n* (%)	86 (71%)	37 (79%)	0.337	71 (69%)	28 (80%)	0.204
ECOG PS > 1, *n*	3 (2.5%)	2 (4.2%)	0.618	11 (11%)	6 (17%)	0.371
Upper tract UC, *n* (%)	53 (44%)	19 (40%)	0.862	47 (45%)	14 (40%)	0.695
Clinical TNM stage, *n*						
T4	51 (42%)	19 (40%)	1.000	51 (49%)	19 (54%)	0.697
*N*+ or M1	109 (89%)	37 (79%)	0.082	100 (96%)	28 (80%)	0.006
Liver meta+, *n* (%)	9 (7.4%)	1 (2.1%)	0.287	11 (11%)	1 (3.0%)	0.295
Local therapy, *n* (%)	48 (39%)	7 (15%)	0.003	44 (42%)	10 (29%)	0.166
Carboplatin‐based regimens, *n*	40 (33%)	20 (43%)	0.282	30 (29%)	14 (40%)	0.293
Number of 1st‐line cycles, *n* (%)			0.051			< 0.001
2 cycles	0	0		29 (28%)	21 (60%)	
3 cycles	0	0		20 (19%)	14 (40%)	
4 cycles	70 (57%)	35 (74%)		46 (44%)	0	
≥ 5 cycles	52 (43%)	12 (26%)		9 (9%)	0	
Best ORR, *n* (%)			0.172			0.225
CR	4 (3.3%)	1 (2.1%)		0	3 (8.6%)	
PR	62 (51%)	23 (49%)		38 (37%)	15 (43%)	
SD	39 (32%)	23 (49%)		58 (56%)	17 (49%)	
Unknown	17 (14%)	0		8 (7.7%)	0	
Subsequent therapy, *n* (%)			< 0.001			0.245
Pembrolizumab	45 (37%)	2 (4.3%)		21 (20%)	3 (8.6%)	
EV	0	13 (28%)		0	8 (23%)	
Death from any cause, *n* (%)	114 (64%)	13 (28%)		84 (80%)	15 (43%)	

### 3.2 Primary Outcomes: Standard‐Switch Cohort

**FIGURE 2:**
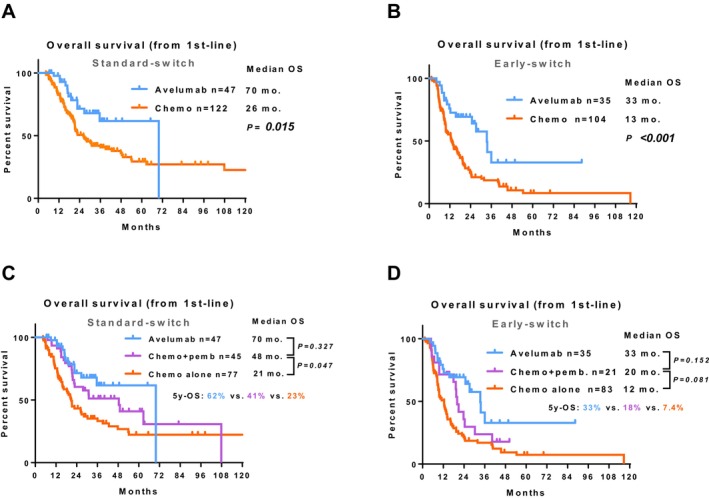
Primary and secondary outcomes of patients with advanced urothelial carcinoma. Comparison of OS between the avelumab and chemotherapy groups in patients with non‐PD at cycle 4 (A) and cycle 2–3 (B). Comparison of OS between the avelumab, chemo + pembrolizumab, and chemotherapy alone groups in patients with non‐PD at cycle 4 (C) and cycle 2–3 (D).

In the standard‐switch cohort (non‐PD at the cycle‐4 assessment), overall survival (OS) measured from first‐line initiation was significantly longer with avelumab than with chemotherapy (median 70 vs. 26 months; *p* = 0.015; Figure 2A).

### 3.3 Secondary Outcomes

In the early‐switch cohort (non‐PD at cycles 2–3), OS from first‐line initiation was significantly longer with avelumab than with chemotherapy (median 33 vs. 13 months; *p* < 0.001; Figure 2B). Restricting the analysis to patients in the chemo cohort who subsequently received second‐line pembrolizumab, OS from first‐line initiation did not differ significantly between the avelumab and chemo groups in the standard‐switch stratum (median 70 vs. 45 months; *p* = 0.327; Figure 2C) or in the early‐switch stratum (median 33 vs. 20 months; *p* = 0.152; Figure 2D).

### 3.4 Multivariable Cox Regression Analysis for the Impact of Avelumab Maintenance Therapy on OS

The background characteristics of patients for this analysis (*n* = 308) are shown in Table 2. The multivariable Cox regression analysis for OS revealed that avelumab (HR 0.37, *p* < 0.001) and subsequent pembrolizumab/EV (HR 0.52, *p* < 0.001) treatments were significantly associated with risk reduction (Table 3). Conversely, poor ECOG PS and < 4 first‐line chemotherapy cycles were significantly associated with an increased risk of OS.

**TABLE 2:** Background of patients for Cox regression analysis.

	Chemo	Avelumab	*p*
Number of patients	226	82	
Age, years (IQR)	72 (64, 78)	73 (66, 80)	0.117
Male, *n* (%)	157 (70%)	65 (79%)	0.114
ECOG PS > 1, *n*	5 (2.2%)	8 (9.8%)	0.007
Upper tract UC, *n* (%)	100 (44%)	33 (40%)	0.603
Clinical TNM stage, *n*			
T4	51 (23%)	19 (23%)	1.000
*N*+ or M1	209 (93%)	65 (79%)	0.002
Liver meta+, *n* (%)	20 (8.8%)	2 (2.4%)	0.077
Local therapy, *n* (%)	92 (41%)	17 (21%)	0.001
Carboplatin‐based regimens, *n*	70 (31%)	34 (42%)	0.102
Number of 1st‐line cycles, *n* (%)			0.024
2 cycles	29 (13%)	21 (26%)	
3 cycles	20 (8.8%)	14 (17%)	
4 cycles	116 (51%)	35 (43%)	
≥ 5 cycles	61 (27%)	12 (15%)	
Best ORR, *n* (%)			1.000
CR	4 (1.8%)	4 (4.9%)	
PR	100 (44%)	38 (46%)	
SD	97 (43%)	40 (49%)	
Unknown	25 (11%)	0	
Subsequent therapy, *n* (%)			< 0.001
Pembrolizumab	66 (29%)	5 (6.1%)	
EV	0	21 (26%)	
Death from any cause, *n* (%)	157 (70%)	28 (34%)	

**TABLE 3:** Multivariable Cox regression analysis for OS (*n* = 308).

	*p*	HR	95% CI
Age, years	0.731	1.00	0.99–1.02
ECOG PS (0–3)	< 0.001	1.54	1.27–1.86
Male	0.908	0.98	0.72–1.35
Treatment era < 2018	0.557	1.13	0.75–1.72
M1 disease	0.202	1.23	0.90–1.67
Local therapy	0.533	0.91	0.66–1.24
1st‐line < 4 cycles	< 0.001	2.21	1.58–3.08
Cisplatin‐based regimen	0.510	1.12	0.80–1.59
Aveluamb	< 0.001	0.38	0.23–0.63
Subsequent pembrolizumab or EV	0.001	0.52	0.36–0.77

### 3.5 Exploratory Outcomes

**FIGURE 3:**
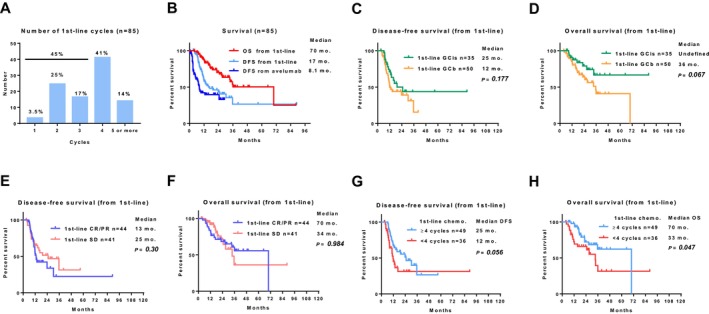
Exploratory outcomes of the 85 patients receiving avelumab maintenance therapy. Number of cycles of first‐line chemotherapy (A). Comparison of OS from first‐line (red line), disease‐free survival (DFS) from first‐line (bright blue), and DFS from avelumab (dark blue) (B). Comparison of DFS (C) and OS (D) between patients treated with 1st‐line GCis and first‐line GCb. Comparison of DFS (E) and OS (F) according to the response rate of the first‐line treatment between the CR/PR and SD groups. Comparison of DFS (G) and OS (H) according to the number of cycles (< 4 vs. ≥ 4 cycles).

Of the 85 patients who underwent avelumab maintenance therapy, 55% received > 4 cycles of first‐line chemotherapy (Table 4 and Figure 3A). The median DFS from avelumab, DFS from first‐line therapy, and OS from first‐line therapy were 8.1, 17, and 70 months, respectively (Figure 3B). First‐line chemotherapy regimens were not significantly associated with DFS (Figure 3C). However, the GCis regimen was significantly associated with OS (Figure 3D). Objective responses to the first‐line chemotherapy were not significantly associated with DFS (Figure 3G) or OS (Figure 3F). Four or more first‐line cycles tended to be associated with DFS (median OS 25 vs. 12 months, *p* = 0.056, Figure 3G) and OS (median OS 70 vs. 33 months, *p* = 0.047, Figure 3H).

**TABLE 4:** Background of patients for exploratory analysis.

	Avelumab
Number of patients	85
Age, years (IQR)	73 (67, 80)
Male, *n* (%)	67 (79%)
ECOG PS > 1, *n*	8 (9%)
Upper tract UC, *n* (%)	33 (39%)
Clinical TNM stage, *n*	
T4	20 (24%)
N+	42 (49%)
M1	43 (51%)
Liver meta+, *n* (%)	2 (2%)
Local therapy, *n* (%)	17 (20%)
Carboplatin‐based regimens, *n*	50 (59%)
Number of 1st‐line cycles, *n* (%)	
2 cycles	21 (25%)
3 cycles	14 (17%)
4 cycles	35 (41%)
≥ 5 cycles	12 (14%)
Best ORR, *n* (%)	
CR	3 (3.5%)
PR	39 (46%)
SD	43 (51%)
Death from any cause, *n* (%)	29 (34%)

## 4 Discussion

Since the JAVELIN Bladder 100 trial established avelumab first‐line maintenance as a standard option for patients without progression after platinum chemotherapy, real‐world evidence has begun to accumulate across healthcare systems. These observational data complement trial findings by reflecting heterogeneity in patient selection, chemotherapy backbones (including frequent carboplatin use), and care delivery. Recent real‐world cohorts in Europe—READY, an Italian compassionate‐use program (*n* = 414) [31], and AVENANCE, a French noninterventional study (*n* = 595) [25]—confirmed the effectiveness and safety of avelumab first‐line maintenance, reporting median OS from avelumab initiation was 26.2 months in READY and 21.3 months in AVENANCE, respectively. However, both were single‐arm studies restricted to patients without progression after 4–6 cycles of platinum and lacked an active comparator, leaving unanswered whether maintenance is superior to continuation of chemotherapy without a treatment break or in patients assessed before completing 4 cycles.

Our multicenter retrospective study addresses these gaps by including a contemporaneous cohort that continued platinum‐based chemotherapy and by stratifying outcomes by chemotherapy exposure (non‐PD at cycles 2–3 and at cycle 4). Avelumab maintenance therapy was associated with longer overall survival (OS) than continued chemotherapy across both treatment groups: 70 versus 26 months (non‐PD at cycle 4) and 33 versus 13 months (non‐PD at cycles 2–3). This head‐to‐head comparison contributes preliminary evidence that can inform decisions when four cycles have not yet been completed. The number of cycles before the maintenance avelumab therapy is worth considering. A randomized phase II trial (DISCUS trial; EudraCT2021‐001975‐17) [32] is currently being conducted to compare the efficacy of switching maintenance therapy after 3 or 6 cycles of first‐line chemotherapy based on the same concept [33]. Our observation may highlight the potential benefit of early switching from chemotherapy to avelumab maintenance therapy.

The comparison of outcomes between patients treated with avelumab and patients treated with pembrolizumab in the chemo group provided insights into the advantages of the two therapies. Because pembrolizumab is a secondary treatment used after the first‐line PD, we expected the prognosis of the pembrolizumab group after first‐line chemotherapy to be worse than the prognosis of the avelumab group for those with non‐PD after the first‐line chemotherapy. However, the difference between first‐line avelumab and second‐line pembrolizumab was not significant. Moreover, the difference was similar during the first 2 years, although OS was favorable in the avelumab group. Some patients will respond to subsequent immunotherapy even if they experience PD during first‐line treatment. In this study, we could not determine the advantages of avelumab maintenance therapy over second‐line pembrolizumab due to the small number of patients.

The OS difference between patients transitioning after 2–3 versus 4 cycles should not be attributed to cycle number alone. In our cohort, earlier transition correlated with poorer ECOG performance status and more T4 disease, indicating confounding by indication and selection related to tolerability, comorbidity, or suboptimal early response. Although avelumab maintenance was associated with longer OS than continued chemotherapy within both strata, patients who switched after 2 to 3 cycles had shorter survival than those who switched after 4 cycles. This observation aligns with our previous report [34], where patients who responded to first‐line chemotherapy had more favorable outcomes with second‐line pembrolizumab. Taken together, these findings suggest that chemosensitivity—particularly an early response within the first 4 cycles—may serve as a useful predictive marker. Clinically, early switching may be reasonable when further chemotherapy is impractical, whereas completing 4 cycles remains appropriate when feasible. Prospective studies are needed to determine whether an early‐switch strategy confers a survival advantage [32].

This study is limited by its retrospective, nonrandomized design and the potential for treatment‐selection bias and residual confounding despite multivariable adjustment. Exact calendar dates marking the end of cycles 2–3 and cycle 4 were not consistently available across centers; therefore, a formal landmark analysis anchored at those time points could not be performed. Although measuring OS from first‐line initiation was intended to reduce immortal time bias related to variable first‐line duration, residual immortal time and time‐varying confounding cannot be excluded. Subsequent therapies, including EV, were imbalanced between groups; EV was included as a covariate, but adjustment for post‐baseline treatments is imperfect and residual confounding may persist. Additional sources of bias include heterogeneity in chemotherapy backbones and assessment schedules, absence of centralized radiologic review, and incomplete capture of certain prognostic factors (e.g., comorbidity/frailty indices, renal function parameters, inflammatory markers, PD‐L1 status). Finally, the avelumab cohort was relatively small, and the long study period may introduce era effects, which together may limit generalizability. Despite these limitations, this study provides valuable insights into the real‐world efficacy of avelumab maintenance therapy. Our objective was not to compare the efficacy of first‐line chemotherapy itself, but to evaluate avelumab maintenance versus continued chemotherapy among patients who achieved disease control (CR/PR/SD) on first‐line therapy. The novelty of our study lies in (i) the inclusion of an active comparator cohort that continued chemotherapy without a treatment interruption and (ii) the analysis of patients assessed after only 2–3 cycles (an early‐switch subgroup). To our knowledge, this subgroup has been underexamined in prior studies, providing an additional perspective. Larger prospective studies are required to obtain more robust evidence regarding the oncological outcomes and the association between the number of cycles and the efficacy of avelumab.

## 5 Conclusion

Avelumab maintenance was associated with improved outcomes across both early‐ and standard‐switch strata in real‐world practice. Careful patient selection may be important for optimizing outcomes.

## Author Contributions

**Noritaka Ishii:** writing – original draft, writing – review and editing, data curation. **Yuya Sekine:** data curation. **Masanao Shinohara:** data curation. **Yohei Kawashima:** data curation. **Kanami Mori:** data curation. **Mizuki Kobayashi:** data curation. **Kazuyuki Numakura:** data curation. **Jotaro Mikami:** data curation. **Naoki Fujita:** data curation. **Teppei Okamoto:** data curation. **Takahiro Yoneyama:** data curation. **Ryuji Tabata:** data curation. **Satoshi Sato:** data curation. **Tomonori Habuchi:** resources, supervision, conceptualization. **Chikara Ohyama:** conceptualization, supervision, resources, funding acquisition. **Shingo Hatakeyama:** conceptualization, investigation, funding acquisition, writing – original draft, writing – review and editing, visualization, methodology, software, formal analysis, project administration, data curation.

## Ethics Statement

This retrospective, multicenter study adhered to the tenets of the Declaration of Helsinki and was approved by the ethics committee of the Hirosaki University School of Medicine (2019‐099‐3 and 2021‐158‐2).

## Consent

Written consent was not obtained, but patients could choose to be excluded from the study (opt‐out approach).

## Conflicts of Interest

Shingo Hatakeyama received honoraria from Janssen Pharmaceutical K.K., Astellas Pharma Inc., AstraZeneca K.K., Ono Pharmaceutical Co. Ltd., Bayer AG, Pfizer Inc., Bristol‐Myers Squibb, Merck Biopharma Co. Ltd., Kaneka Corporation, and Nipro Corporation. The other authors have no conflicts of interest to declare.

## Data Availability

The data that support the findings of this study are available from the corresponding author upon reasonable request.
